# Schizophrenia and obesity: May the gut microbiota serve as a link for the pathogenesis?

**DOI:** 10.1002/imt2.99

**Published:** 2023-04-04

**Authors:** Hui Wu, Yaxi Liu, Jie Wang, Shengyun Chen, Liwei Xie, Xiaoli Wu

**Affiliations:** ^1^ Psychiatry Department Third Affiliated Hospital of Sun Yat‐sen University Guangzhou China; ^2^ State Key Laboratory of Applied Microbiology Southern China, Guangdong Provincial Key Laboratory of Microbial Culture Collection and Application, Institute of Microbiology Guangdong Academy of Sciences Guangzhou China; ^3^ Department of Life Sciences Imperial College London London United Kingdom

**Keywords:** gut microbiota, obesity, prebiotics, probiotics, schizophrenia

## Abstract

Schizophrenia (SZ) places a tremendous burden on public health as one of the leading causes of disability and death. SZ patients are more prone to developing obesity than the general population from the clinical practice. The development of obesity frequently causes poor psychiatric outcomes in SZ patients. In turn, maternal obesity during pregnancy has been associated with an increased risk of SZ in offspring, suggesting that these two disorders may have shared neuropathological mechanisms. The gut microbiota is well known to serve as a major regulator of bidirectional interactions between the central nervous system and the gastrointestinal tract. It also plays a critical role in maintaining physical and mental health in humans. Recent studies have shown that the dysbiosis of gut microbiota is intimately associated with the onset of SZ and obesity through shared pathophysiological mechanisms, particularly the stimulation of immune inflammation. Therefore, gut microbiota may serve as a common biological basis for the etiology in both SZ and obesity, and the perturbed gut–brain axis may therefore account for the high prevalence of obesity in patients with SZ. On the basis of these findings, this review provides updated perspectives and intervention approaches on the etiology, prevention, and management of obesity in SZ patients by summarizing the recent findings on the role of gut microbiota in the pathogenesis of SZ and obesity, highlighting the role of gut‐derived inflammation.

## Highlights

- Obesity represents a major challenge to the clinical management of schizophrenia (SZ) and contributes to worse psychiatric outcomes in SZ.
- Perturbations in gut–brain axis, particularly gut‐derived inflammation, are closely related to the pathogenesis of both SZ and obesity, suggesting that gut microbiota may be a potential hub linking these two disorders.
- Gut microbiota is a modifiable component that may be used as a novel therapeutic option for treating obesity or improving psychiatric outcomes in SZ patients.
- Future research on obesity in SZ includes understanding the therapeutic potential of the gut microbiota, its temporospatial dynamics, its mechanistic interaction with the host, and the crosstalk between the gut and the brain.

## Graphical abstract

This review provides updated perspectives on the etiology, prevention, and management of obesity in SZ patients by summarizing the recent findings on the role of gut microbiota in the pathogenesis of SZ and obesity, highlighting the role of gut‐derived inflammation.

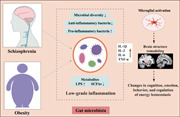

## INTRODUCTION

Schizophrenia (SZ) is a chronic and severe psychiatric disorder with a lifetime prevalence of approximately 1% worldwide [1] and 0.6% in China [2]. SZ is defined as abnormalities in cognition, thinking, emotion, and behavior as well as incompatibility between mental activities and the external environment. It often appears at the age of adolescence and early adulthood. SZ patients usually face challenging circumstances as a result of a high recurrence rate, recurrent disabilities, extreme poverty, and a low treatment effectiveness, placing a significant burden on individuals, families, and societies [3].

Notably, a significant proportion of SZ patients experienced early onset of metabolic syndrome (MS), which worsened as the disease progresses [4, 5]. Among them, obesity is the most common characteristics of the MS in SZ. On the basis of a 6‐month follow‐up assessment, the prevalence of obesity in SZ was reported to be approximately 20% and with progression of the conditions, this prevalence could rise to 60% or even higher, which is significantly higher than the general population [6]. Clinically, obesity is associated with adverse psychiatric outcomes in SZ, in addition to poor physical health and a lower life expectancy [7, 8]. Moreover, the development of obesity has been shown to be linked to brain anatomical abnormalities in both healthy individuals [9] and patients with SZ [10, 11]. Importantly, all these obesity‐related brain regions are frequently disrupted in SZ [12], suggesting that two disorders may have shared neuropathological and pathophysiological mechanisms.

**Figure 1:**
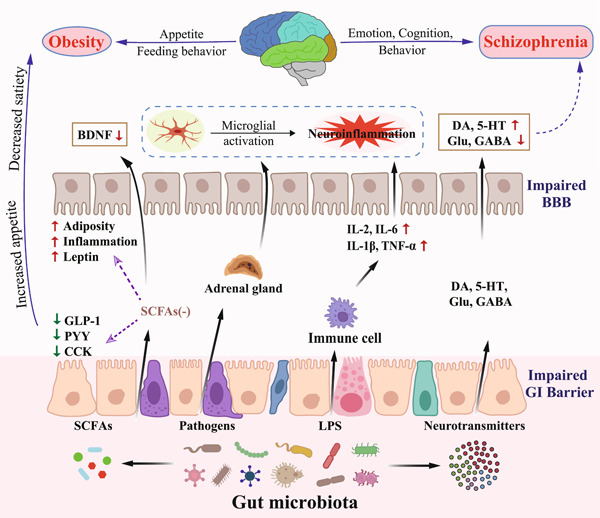
Role of gut microbiota in the pathophysiology of schizophrenia and obesity. The gut microbiota can convert dietary nutrients into metabolites, such as SCFAs, and neurotransmitters, such as DA, 5‐HT, Glu, and GABA. These metabolites have different peripheral and central effects that can alter host cognition, mood and behavior on the one hand, and modify host metabolism and central regulation of appetite on the other hand. The realization of these processes includes the direct crossing of the BBB, the stimulation of the afferent vagus nerve and the triggering of the intestinal endocrine/immune system and the HPA axis, ultimately leading to microglial activation and thus stimulation of upstream neural circuits. 5‐HT, serotonin; BBB, blood–brain barrier; BDNF, brain‐derived neurotrophic factor; CCK, cholecystokinin; DA, dopamine; GABA, *γ*‐aminobutyric acid; GI, gastrointestinal; GLP‐1, glucagon‐like peptide‐1; Glu, glutamate; IL‐1*β*, interleukin 1*β*; IL‐2, interleukin 2; IL‐6, interleukin 6; LPS, lipopolysaccharide; PYY, peptide YY; SCFAs, short‐chain fatty acids; TNF‐*α*, tumor necrosis factor *α*.

The “gut–brain axis” research and findings have laid the foundation for the interaction between gut microbiota and host health in recent years. Emerging evidence indicates that the dysbiosis of gut microbiota may play a significant role in the emergence of SZ and obesity. First, gut microbiome is essential for normal metabolic and immunological function of the host. Furthermore, perturbations in the gut microbiota have been reported to be linked to the pathogenesis of obesity by influencing glucose/lipid tolerance, insulin resistance, and low‐grade inflammation [13]. The pathophysiology of SZ has also been linked to similar processes [14]. Second, the dysbiosis of gut microbiota may promote the release of circulating proinflammatory cytokines, and some of which may pass through the blood–brain barrier (BBB) to cause neuroinflammation, such as microglia proliferation, which is associated with altered brain neurological substrates in both SZ and obesity [15]. In addition, gut microbial dysbiosis could also lead to the activation of the hypothalamic‐pituitary‐adrenal (HPA) axis, resulting in elevated circulating cortisol levels and decreased levels of brain‐derived neurotrophic factor [16], both of which are also associated with lower brain volume and poor cognitive performance in both patients with SZ and obesity. On the basis of the aforementioned theoretical framework, we propose that the high prevalence of obesity in SZ may be related to shared pathophysiological pathways, and that gut microbial dysbiosis may provide a common biological basis for the etiology of both SZ and obesity (Figure 1).

## GUT–BRAIN AXIS AND GUT MICROBIOTA

The gut–brain axis is a bidirectional information exchange system that links the gastrointestinal (GI) track and brain. The gut microbiota is considered to be a key regulator of this axis, forming the Microbiota–Gut–Brain (MGB) axis [17]. On the one hand, gut microbiota can affect brain development through neural, immune, and endocrine pathways, regulating mood, cognition, and behavioral phenotypes of the host; on the other hand, the brain can also regulate GI function and homeostasis through neural and endocrine pathways, influencing the composition, structure, and function of gut microbiota.

Nearly 100 trillion microorganisms exist in human GI track. These microorganisms are significant to the development of the immune system, and are required for the host to maintain metabolic and physiological homeostasis [18, 19]. The intestinal microorganism is mainly composed of bacteria, followed by a smaller proportion of fungi, viruses, and archaea. The gut microbiota may be divided into six major phyla based on the metagenomic sequencing, including *Firmicutes*, *Bacteroidetes*, *Proteobacteria*, *Actinobacteria*, *Verrucomicrobia*, and *Fusobacteria* [20]. Among them, the two most prominent phyla are *Firmicutes* and *Bacteroidetes*, which account for about 70%–75% of the total gut microbiota. In addition, researchers have proposed three different enterotypes to classify the gut microbial community into different enterotypes, that is, enterotype of *Bacteroides*, *Prevotella*, and *Ruminococcus* [21]. Each enterotype is distinguished by relatively high levels of particular microbial genera that are functional relevance. For example, the enterotype‐*Bacteroides* is associated with a chronic high‐fat or high‐protein diet, while the enterotype‐*Prevotella* is associated with a high‐carbohydrate diet [22]. The proposal of these enterotypes may help us gain a better knowledge of how the distribution, composition, and structure of the gut microbiota impact human health and illness, notwithstanding the criticism surrounding this categorization system [23].

The gut microbiota is also dynamically influenced by a variety of factors, such as genetics, diet, metabolism, age, geography, antibiotic therapy, and stress [24, 25, 26]. Therefore, the structure of gut microbiome is also a good characterization of individual environmental history, leading to individual differences in disease risk, disease progression, and response to treatment. As an important internal environmental factor, gut microbiota is directly implicated in various pathophysiological processes of the host, including cytokine release, neurotransmitter production, tryptophan metabolism, and oxidative stress. This is accomplished by stimulating the upstream neural route via the vagus nerve, triggering the enteroendocrine/immune system and the HPA axis [27]. Additionally, the gut microbiota may also be involved in various pathophysiological processes associated with the disease onset and progression by affecting host gene expression or epigenetic modifications [28].

## SCHIZOPHRENIA

Although the pathogenesis of SZ remains unclear, the interaction between genetic and environmental risk factors has been proposed as the potential cause leading to the development of SZ. Among them, the genetic basis determines the susceptibility of an individual, while environmental factors could be the initiating factors that determine whether an individual develops the disease or not. In the development and progression of SZ, the gut microbiota is also considered to be one of the key internal environmental factors.

### Gut microbiota in SZ

**Figure 2:**
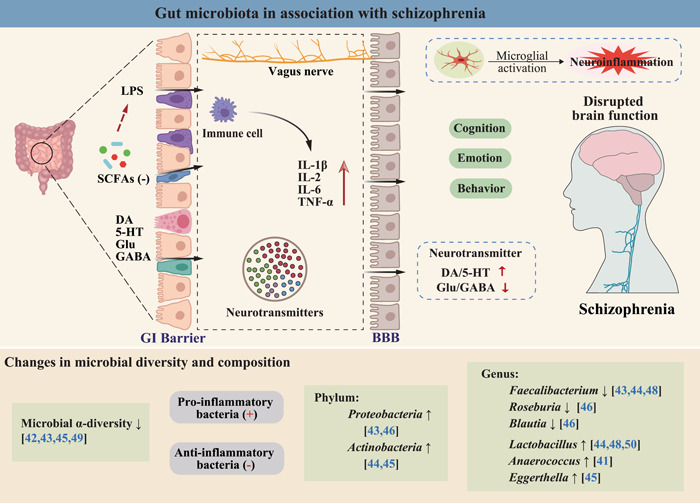
Gut microbiota in association with schizophrenia. 5‐HT, serotonin; BBB, blood–brain barrier; DA, dopamine; GABA, *γ*‐aminobutyric acid; GI, gastrointestinal; Glu, glutamate; IL‐1*β*, interleukin 1*β*; IL‐2, interleukin 2; IL‐6, interleukin 6; LPS, lipopolysaccharide; SCFAs, short‐chain fatty acids; TNF‐*α*, tumor necrosis factor *α*.

There are numerous hypotheses for the pathogenesis of SZ, that is, the dopamine (DA) hypothesis, the glutamate (Glu) hypothesis, the 5‐hydroxytryptamine (5‐HT) hypothesis, the *γ*‐aminobutyric acid (GABA) hypothesis, and the dysconnection hypothesis [29, 30]. The DA hypothesis is one of those that is commonly considered to contribute to the etiology of SZ. This is demonstrated by the observation of the overactive dopaminergic neurons in patients with SZ and the evidence that most antipsychotic drugs reduce the psychiatric symptoms by blocking dopaminergic receptors. However, no single hypothesis can explain the complexity of SZ. In recent years, the association of gut microbiota with many psychiatric disorders has been recognized. Thus, the complexity of SZ can be comprehensively explained by the MGB axis, which links different hypotheses together (Figure 2).

The dysregulation of several neurotransmitters, for example, the hyperactivation of DA and 5‐HT, an insufficient level of Glu and GABA in the brain, may contribute to the onset of SZ [31]. According to recent findings, the BBB and intestinal barrier are structurally and functionally comparable, and bioactive compounds originating from the gut microbiota can cross both barrier systems similarly, impacting brain function [32]. In fact, more than 90% of 5‐HT in the human body is synthesized in the intestine, and the gut microbiota contributes to the biosynthesis of 95% of 5‐HT in the colonic enterochromaffin cell. Additionally, the gut microbiota can release functional neurotransmitters (e.g., the DA, Glu, 5‐HT, and GABA), via digestion and breakdown of compounds from food. Some of these neurotransmitters can cross the BBB directly to influence the function of the central nervous system [33]. Furthermore, metabolites derived from the gut microbiota, such as indoles and short‐chain fatty acids (SCFAs), can influence host cognition, mood, and behavior directly or indirectly through their neuroactive properties and effects on other gut–brain signaling pathways, such as the immune and endocrine systems [34, 35, 36].

Moreover, the association between SZ and MS has become an emerging issue to be addressed in the management of SZ. According to the clinical data from our group, patients with first‐episode SZ exhibited glucolipid metabolic abnormalities before receiving antipsychotic medication [37]. The use of antipsychotics further worsened the insulin resistance and related metabolic disorders in SZ patients [38]. Moreover, the discontinuation of antipsychotic medication was not associated with improvement of glucolipid metabolic disturbance in these patients [39]. These findings suggest that SZ itself may involve pathophysiological processes that affect the body's glucolipid metabolism. Of note, existing studies suggest that gut microbiota and its metabolites can affect the body's glucolipid metabolism by regulating host gut hormone secretion, immunological inflammation, and insulin sensitivity [13]. Thus, dysbiosis of the gut microbiota is also thought to be associated with the high prevalence of MS in SZ.

### Evidence of disturbed gut microbiota from clinical studies

The dysbiosis of gut microbiota in patients with SZ has been documented in numerous clinical studies. Initially, a metagenomic sequencing analysis showed that the oropharyngeal microbiota of schizophrenic and healthy populations differed dramatically at the phylum and genus levels [40]. Subsequently, several studies compared the differences in gut microbial composition between schizophrenic and healthy populations. These results indicated that the diversity and composition of the gut microbiota were significantly altered in patients with SZ [41, 42, 43, 44, 45] and that specific bacteria may serve as biomarkers to distinguish SZ patients from healthy populations [46, 47]. Additionally, the severity of psychiatric symptoms and responsiveness to subsequent treatment in first‐episode SZ patients were strongly correlated with the relative abundance of *Lactobacillus* [48]. Moreover, the gut microbiota of SZ patients differed significantly between the acute and remission phases [47, 49]. Patients with acute exacerbation exhibited higher abundance of *Bacteroides* and lower abundance of *Prevotella* than those in remission, which was strongly correlated with the severity of their psychiatric symptoms [49]. Likewise, the gut microbiota differed significantly between patients with first‐episode drug‐naïve and those with chronic medication [50], suggesting that antipsychotics may also impact and reshape the gut microbial profiles. Furthermore, Zheng et al. [42] found that transplantation of fecal microbiota from SZ patients into germ‐free mice reduced Glu levels in the hippocampus of the mice, suggesting that the gut microbiota itself can influence the brain neurochemistry associated with the onset of SZ. Similarly, transplantation of fecal microbiota from drug‐free patients with SZ into specific pathogen‐free mice has been reported to induce SZ‐like behaviors [51]. Of note, these mice also showed an elevation in the kynurenine‐kynurenic acid pathway of tryptophan degradation in both periphery and brain, suggesting that changes in the gut microbial composition may contribute to the onset of SZ by manipulating tryptophan‐kynurenine metabolism [51]. Together, these results suggest that alterations in the gut microbiota may be an important driver in the development of SZ (Table 1).

**Table 1:** Summary of schizophrenia‐related gut microbial alterations reported in clinical studies.

Author (Year)	Study design	Population characteristics	Intervention	Clinical findings (vs. HCs)
*Evidence for: Low‐grade inflammation mediated by gut microbial dysbiosis*				
Zheng et al. (2019) [42]	Cross‐sectional study Fecal samples 16S rRNA sequencing	Chinese population: 63 SZ, 69 HCs Female: 33%, 48% Age (years): 43.49 ± 1.68, 39.99 ± 1.62 BMI (kg/m^2^): 22.90 ± 0.32, 23.16 ± 0.33 Illness duration (N/A) Antipsychotics (58 SZ)	N/A	Microbial α‐diversity ↓ *Veillonellaceae* and PANSS scores (−) *Lachnospiraceae* and PANSS scores (+) Microbial panel (*Aerococcaceae*, *Bifidobacteriaceae*, *Brucellaceae*, *Pasteurellaceae*, and *Rikenellaceae*) for discriminating SZ from HCs (ROC = 0.769)
Zhang et al. (2020) [43]	Cross‐sectional study Fecal samples 16S rRNA sequencing	Chinese population: 10 FSZ, 16 HCs Female: 40%, 44% Age (years): 37.6 ± 7.2, 35.8 ± 6.8 BMI (kg/m^2^): 23.3 ± 6.8, 22.3 ± 6.5	N/A	Microbial α‐diversity ↓ Phylum: *Proteobacteria* ↑ Genus: *Faecalibacterium* and *Lachnospiraceae* ↓
Li et al. (2020) [44]	Cross‐sectional study Fecal samples 16S rRNA sequencing	Chinese population: 82 SZ, 80 HCs Female: 44%, 51% Age (years): 42.15 ± 13.13, 41.03 ± 14.34 BMI (kg/m^2^): 24.48 ± 4.33, 23.03 ± 3.05 Illness duration (N/A) Antipsychotics (75 SZ)	N/A	Microbial α‐diversity (ns) Phylum: *Actinobacteria* ↑; *Firmicutes* ↓ Genus: *Collinsella*, *Lactobacillus*, *Succinivibrio*, *Mogibacterium*, *Corynebacterium* ↑; *Adlercreutzia*, *Anaerostipes*, *Ruminococcus* and *Faecalibacterium* ↓. *Succinivibrio* and PANSS scores (+) *Corynebacterium* and PANSS scores (−)
Xu et al. (2020) [45]	Cross‐sectional study Fecal samples Shotgun sequencing 16S rRNA sequencing	Chinese population: Discovery: 40 SZ, 40 HCs Female: 50%, 50%. BMI: N/A Age (years): 35 ± 11, 34 ± 9 Validation: 44 SZ, 44 HCs Female: 36%, 36% Age (years): 35 ± 11, 35 ± 11 BMI (kg/m^2^): 22.0 ± 3.2, 23.1 ± 3.7 Illness duration (N/A) Antipsychotics (N/A)	N/A	Microbial α‐diversity ↓ Phylum: *Actinobacteria* ↑ Genus: *Eggerthella* and *Megasphaera* ↑; *Enterococcus* ↓ Species: *Akkermansia muciniphila*, *Bifidobacterium adolescentis*, *Clostridium perfringens*, *Lactobacillus gasseri* and *Megasphaera elsdeniis*↑
Shen et al. (2018) [46]	Cross‐sectional study Fecal samples 16S rRNA sequencing	Chinese population: 64 SZ, 53 HCs Female: 44%, 34% Age (years): 42 ± 11, 39 ± 14 BMI (kg/m^2^): 23.49 ± 3.8, 23.14 ± 2.8 Illness duration ≤ 10 years Antipsychotics >6 months	N/A	Microbial α‐diversity (ns) Phylum: *Proteobacteria* **↑** Genus: *Succinivibrio*, *Megasphaera*, *Collinsella*, *Clostridium*, *Klebsiella*, *Methanobrevibacter* ↑; *Blautia*, *Coprococcus*, *Roseburia* **↓**. Microbial panel discriminating SZ from HCs (ROC = 0.837)
Schwarz et al. (2018) [48]	Cross‐sectional and 12 month follow‐up Fecal samples Shotgun sequencing	Finnish population: 28 FEP, 16 HCs Female: 43%, 50% Age (years): 25.9 ± 5.5, 27.8 ± 6.0 BMI (kg/m^2^): 23.8 ± 4.3, 23.9 ± 3.1	N/A	Genus: *Lactobacillus*, *Tropheryma*, *Halothiobacillus*, *Saccharophagus*, *Ochrobactrum*, *Deferribacter* and *Halorubrum* ↑; *Anabaena*, *Nitrosospira*, *Gallionella*, *Bacteroides*, *Ruminococcus*, and *Faecalibacterium* **↓** *Lactobacillus* and psychotic symptoms (+) *Lactobacillus* and treatment response (−) (up to 12 months)
Zhang et al. (2018) [49]	Cross‐sectional study Fecal samples 16S rRNA sequencing	Chinese population: 12 aSZ, 13 rSZ Female: 50%, 46% Age (years): 36.5 ± 8.9, 36.2 ± 8.2 BMI (kg/m^2^): 23.7 ± 3.2, 24.2 ± 2.7 Duration (years): 8.0 ± 6.9, 9.3 ± 6.4 Antipsychotics >6 months	N/A	aSZ (vs. rSZ): Microbial α‐diversity ↓ Genus: *Bacteroides* ↑, *Prevotella* ↓ Genus *Prevotella* and PANSS scores (−)
*Evidence against: Low‐grade inflammation mediated by gut microbial dysbiosis*				
Nguyen et al. (2018) [41]	Cross‐sectional study Fecal samples 16S rRNA sequencing	American population: 25 SZ, 25 HCs Female: 40%, 44% Age (years): 52.9 ± 11.2, 54.7 ± 10.7 BMI (kg/m^2^): 31.8 ± 5.4, 28.9 ± 4.0 Illness duration >10 years Antipsychotics (21 SZ)	N/A	Microbial α‐diversity (ns) Phylum: *Proteobacteria* ↓ Genus: *Anaerococcus* ↑; *Haemophilus*, *Sutterella*, *Clostridium* ↓. Family *Ruminococcaceae* and Negative symptoms (−)
Ma et al. (2020) [50]	Cross‐sectional study Fecal samples 16S rRNA sequencing	Chinese population: (40 FSZ, 85 TSZ), 69 HCs Female: 46%, 46% Age (years): 24.19 ± 6.18, 23.14 ± 3.20 BMI: 18–25 kg/m^2^ Antipsychotics (TSZ) > 3 months Illness duration (TSZ) > 1 year	N/A	FSZ (vs. HCs): Microbial α‐diversity (ns) Genus: *Actinobacillus*, *Fusobacterium*, *Megasphaera*, *SMB53* ↓; *Escherichia* ↑ TSZ (vs. FSZ and HCs): Microbial α‐diversity ↓ Genus: *Escherichia*, *Enterococcus*, *Lactobacillus*, *Shigella*, *Streptococcus* ↑

Abbreviations: aSZ, acute schizophrenia; BMI, body mass index; FEP, first‐episode psychosis; FSZ, first‐episode drug‐naïve schizophrenia; HCs, healthy controls; PANSS, positive and negative syndrome scale; ROC, receiver operating characteristic; rRNA, ribosomal RNA; rSZ, remission schizophrenia; SZ, schizophrenia; TSZ, chronically antipsychotic‐treated schizophrenia.

Treatment resistance in SZ may also be related to the MGB axis. In clinical practice, although most patients with SZ respond well to available treatment regimens, approximately 30% of patients still respond poorly to both typical and atypical antipsychotics and exhibit persistent psychotic symptoms, a condition known as treatment‐resistant schizophrenia (TRS) [52]. The mechanisms underlying treatment resistance are still unknown, but one study found that the addition of minocycline treatment improved working memory, lack of volition, and anxiety‐depression symptoms in patients with TRS [53]. Therefore, it is speculated that treatment resistance may also be related to the MGB axis. However, it is important to note that while the effects on other symptoms (e.g., depression and anxiety) in TRS patients through modulation of the gut microenvironment are promising, the available evidence for the effects of probiotics on their psychiatric symptoms is scarce and unsatisfactory [54], making it difficult to draw conclusions. In a double‐blind randomized controlled trial on individuals with SZ, treatment with *Lactobacillus* and *Bifidobacterium* did not result in improved psychiatric symptoms than the placebo group [55]. The reasons for this negative result may come from two sources: on the one hand, the role of gut microbiota in the pathogenesis of SZ has not been revealed at the strain level, that is, whether the pathogenesis of SZ is associated with specific strains of bacteria or the pooled effect of multiple microbiota; on the other hand, the physical structure and neural substrate of the brain have been irreversibly altered in schizophrenic patients, and the alterations cannot be reversed simply by modulating the gut microbiota.

## OBESITY

### Gut microbiota in obesity

**Figure 3:**
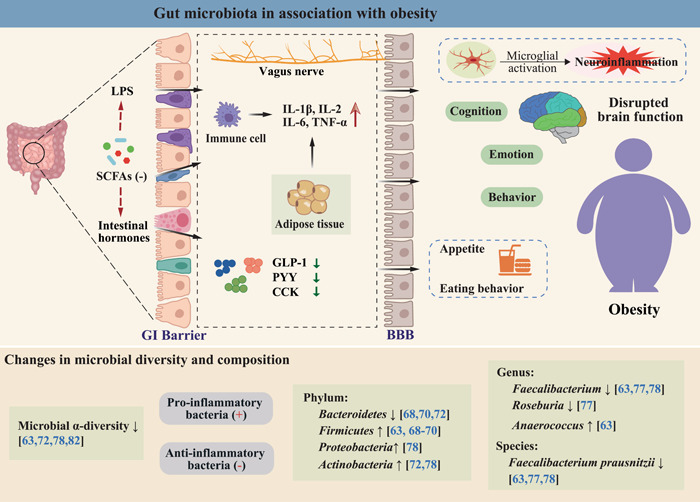
Gut microbiota in association with obesity. BBB, blood–brain barrier; CCK, cholecystokinin; GI, gastrointestinal; GLP‐1, glucagon‐like peptide‐1; IL‐1*β*, interleukin 1*β*; IL‐2, interleukin 2; IL‐6, interleukin 6; LPS, lipopolysaccharide; PYY, peptide YY; SCFAs, short‐chain fatty acids; TNF‐*α*, tumor necrosis factor *α*.

The etiology of obesity involves a complex interplay of host genetics, social environment, dietary choices, and psychosomatic variables. Despite the established link between the development of obesity and an imbalance of energy intake and expenditure, the maintenance of the body's energy homeostasis is highly regulated by complex and coordinated hormones in the gut (Figure 3). Multiple hormones have contributed to the regulation of energy balance, including leptin receiving feedback from the adipocyte itself, ghrelin secreted by the gastric mucosa, and several brain–gut peptides that regulate appetite, such as glucagon‐like peptide‐1 (GLP‐1), peptide YY, and cholecystokinin [56]. The gut microbiota is an important internal environmental factor that regulates host metabolism, intestinal hormone secretion, immune inflammatory status, insulin sensitivity, and food intake, all processes that are closely associated with the development of obesity [56]. On the one hand, the gut microbiota is involved in food digestion and nutrient absorption, and is highly influenced by dietary patterns and food preferences [57]. On the other hand, the gut microbiota can convert dietary nutrients into various metabolites, such as SCFAs and indoles, and these microbiota‐derived metabolites could further regulate appetite and feeding behavior of the host after being absorbed into the blood [58, 59]. In addition, the gut microbiota can affect the energy balance by altering the capacity for energy acquisition and storage [60, 61]. Therefore, the gut microbiota is considered to be a key regulator during the development of obesity.

### Evidence of disturbed gut microbiota from clinical studies

High‐fat diets have long been recognized to be associated with an increased prevalence of obesity. Notably, high‐fat diets induce weight gain along with negative impact on the gut microbiota via alteration on its composition, diversity and structure [62, 63, 64]. Generally, a high *α*‐diversity is regarded as a sign of “good” health, while loss of diversity occurs in most pathogenic conditions, such as obesity and type 2 diabetes [65, 66]. In addition, obese individuals have shown significant alterations in the composition of the gut microbiota. Early in 2005, a pioneering study revealed decreased phylum *Bacteroidetes* and increased phylum *Firmicutes* in genetically obese mice, thus positing a role of microbial dysbiosis in obesity [67]. Subsequently, this finding was also confirmed in humans, that is, a more widespread *Bacteroides* was found in individuals with normal weight, while an increased abundance of the *Firmicutes* was found in obese individuals [68, 69, 70, 71, 72]. However, other studies have reported conflicting results, suggesting bacterial composition at phylum level cannot be recapitulated in different studies and various conditions. These reports included an increase of Bacteroidetes in overweight individuals [73], an increase of *Bacteroidetes* and a decrease of *Firmicutes* in obese individuals [74], and no difference in the two major phyla (*Bacteroidetes* and *Firmicutes*) between obese and lean individuals [75]. Importantly, the obesity‐associated microbiota has been shown to be more capable of acquiring and storing energy than that of their lean counterparts [60, 61], and this phenotype can be transferred through fecal microbiota transplants (FMTs) [60], resulting in the rapid weight gain in recipient mice even under diet with low fat. This indicates that changes in the gut microbiota may not simply be a consequence of obesity but may have an active role in its pathogenesis.

Moreover, the enrichment of proinflammatory bacteria (e.g., *Enterobacter cloacae*, *Prevotella* genus) and the depletion of anti‐inflammatory bacteria (e.g., *Clostridium* species) have been constantly reported in obese individuals [76, 77, 78]. An imbalance of typical proinflammatory Gram‐negative bacteria and anti‐inflammatory SCFA‐producing bacteria might be a potential driver for the chronic low‐grade inflammation seen in obesity [79, 80, 81]. Nevertheless, this concept has only been confirmed in animal studies and in associative nature in humans, and strong evidence is still lacking. Additionally, patients with severe obesity even showed dysbiosis in gut microbial function, and the success of bariatric surgery in these patients has increasingly been shown to be associated with improved function of the major gut microbiota [82, 83]. Of note, changes in microbial function are also closely related to altered microbial composition, as Turnbaugh et al. [72] found that the microbiota enriched with *Bacteroidetes* had higher levels of functional diversity, while those enriched in *Firmicutes* had lower levels of functional diversity. Taken together, these findings suggest that dysbiosis of the gut microbiota may be an important driver in the development of obesity (Table 2).

**Table 2:** Summary of obesity‐related gut microbial alterations reported in clinical studies.

Author (year)	Study design	Population characteristics	Intervention	Clinical findings (vs. lean/nonobese or baseline)
*Evidence for: Low‐grade inflammation mediated by gut microbial dysbiosis*				
Andoh et al. (2016) [63]	Cross‐sectional study Fecal samples 16S rRNA sequencing	Japanese population: 10 obese, 10 lean Female: 50%, 50% Mean age (years): 41, 45 BMI (kg/m^2^): 38.1 ± 3.5, 16.6 ± 1.0	N/A	Microbial α‐diversity ↓ Phylum: *Firmicutes* ↑; *Fusobacteria* ↑ Genus: *Alistipes*, *Anaerococcus*, *Coprococcus*, *Fusobacterium*, and *Parvimonas* ↑; *Bacteroides*, *Faecalibacterium*, *Desulfovibrio*, *Lachnoanaerobaculum*, *Olsenella* ↓ Anti‐inflammatory bacteria species: *Faecalibacterium prausnitzii* ↓ Proinflammatory bacteria species: *Bacteroides vulgatus* ↑
Turnbaugh et al. (2009) [72]	Comparative study Fecal samples 16S rRNA sequencing Shotgun sequencing	European or African Female: 31 monozygotic twin pairs, 23 dizygotic twin pairs. Age range: 21–32 years Concordant for leanness (BMI: 18.5–24.9 kg/m^2^) or obesity (BMI ≥ 30 kg/m^2^)	N/A	Microbial α‐diversity ↓ Phylum *Firmicutes* (ns) Phylum *Bacteroidetes* ↓ Phylum *Actinobacteria* ↑ *F*/*B* ratio ↑
Haro et al. (2015) [77]	Clincal trial Fecal samples 16S rRNA sequencing	20 obese men: Age: 63.3 ± 2.0 years BMI: 32.2 ± 0.5 kg/m^2^	Two dietary interventions: ① Med (*n* = 10) ② LFHCC (*n* = 10) duration: 1 year	Med diet: Genus: *Prevotella* *↓*; *Roseburia*, *Oscillospira* *↑* Species: *Parabacteroides distasonis* ↑ LFHCC diet: Genus: *Prevotella* *↑*; *Roseburia* *↓* Species: *Faecalibacterium prausnitzii* ↑
Lee et al. (2019) [78]	Clincal trial Fecal samples 16S rRNA sequencing	12 obese women: Age: 52.5 (32–62) years BMI: 37.0 (31.0–40.5) kg/m^2^	BS: ① MWL (*n* = 4) ② AGB (*n* = 4) ③ RYGB (*n* = 4)	RYGB (at 10% weight‐loss): Microbial α‐diversity ↑ Phylum: *Proteobacteria* ↑; *Actinobacteria* ↑ Species*: Faecalibacterium prausnitzii* ↑
Aron‐Wisnewsky et al. (2019) [82]	Clinical trial Fecal samples Shotgun sequencing	61 severe obese women: Age: 36.9 ± 9.86 years BMI: 45.6 ± 5.23 kg/m^2^	BS: ① AGB (*n* = 20) ② RYGB (*n* = 41)	Baseline: Microbial gene richness ↓ 1‐year postsurgery: Microbial gene richness ↑
*Evidence for: Increased phylum Firmicutes and/or decreased phylum Bacteroidetes (increased F/B ratio)*				
Koliada et al. (2017) [68]	Cross‐sectional study Fecal samples 16S rRNA sequencing	Ukrainian population: 11 obese, 16 overweight 27 normal, 7 underweight Female: 75% Mean age: 44.2 years	N/A	Phylum *Firmicutes* ↑ Phylum *Bacteroidetes* ↓ *F*/*B* ratio ↑
Rahat‐Rozenbloom et al. (2014) [69]	Observational study Faecal samples Rectal dialysis bag 16S rRNA sequencing	University of Toronto: 11 overweight, 11 lean Female: 45%, 45% Age (years): 42.5 ± 3.9, 35.8 ± 4.2 BMI (kg/m^2^): 30.1 ± 0.8, 22.6 ± 0.6	N/A	Phylum *Firmicutes* ↑ Phylum *Bacteroidetes* (ns) *F*/*B* ratio ↑
Ley et al. (2006) [70]	Clinical trial Fecal samples 16S rRNA sequencing	12 obese Age range: 21–65 years BMI range: 30–43 kg/m^2^ 2 lean (as blank control) Mean BMI: 23 kg/m^2^ Mean age: 34 years	Weight‐loss diets: ① FAT‐R (*n* = 6) ② CARB‐D (*n* = 6) duration: 1 year	Baseline (obese vs. lean): Phylum *Firmicutes* ↑ Phylum *Bacteroidetes* ↓ *F*/*B* ratio ↑ 1‐year diet‐treatment: Phylum *Firmicutes* ↓ Phylum *Bacteroidetes* ↑ *F*/*B* ratio ↓
*Evidence against: Increased phylum Firmicutes and/or decreased phylum Bacteroidetes (increased F/B ratio)*				
Schwiertz et al. (2010) [73]	Cross‐sectional study Fecal samples 16S rRNA sequencing	University of Giessen and Marburg: 30 obese, 35 overweight, 33 lean Female: 65%. Age: 47 ± 13 years	N/A	Proportion of *Firmicutes* ↓ Proportion of *Bacteroidetes* ↑ *F*/*B* ratio ↓
Kellerer et al. (2019) [74]	Clincal trial Fecal samples 16S rRNA sequencing	German population: 17 morbidly obese, 17 nonobese Female: 82%, 82% Age (years): 41.8 ± 9.1, 41.7 ± 9.7 BMI (kg/m^2^): 52.5 (47.0–56.8), 21.5 (19.6–23.3). BMI‐LSG: 39.1 (32.6–44.0) kg/m^2^ Nonobese as blank control	BS: LSG Follow‐up: 6‐month	Baseline (obese vs. nonobese): Phylum *Firmicutes* ↓ Phylum *Bacteroidetes* ↑ *F*/*B* ratio ↓ LSG (6‐month follow‐up): Phylum *Firmicutes* ↑ Phylum *Bacteroidetes* ↓ *F*/*B* ratio ↑
Duncan et al. (2008) [75]	Clincal trial Fecal samples 16S rRNA sequencing	North of Scotland: 29 obese men (BMI > 30 kg/m^2^) 14 nonobese (BMI < 30 kg/m^2^) Balanced cross‐over design: (on 23 obese individuals)	Weight‐loss diets: ① LC for 4 weeks ② MC for 4 weeks duration: 8 weeks	Cross‐sectional and clincal trial: Phylum *Firmicutes* (ns) Phylum *Bacteroidetes* (ns)

Abbreviations: AGB, adjustable gastric banding; BMI, body mass index; BS, Bariatric surgery; CARB‐R, carbohydrate‐restricted; FAT‐R, fat‐restricted; LC, high‐protein low carbohydrate, ketogenic; LFHCC, a low‐fat, high‐complex carbohydrates diet; LSG, laparoscopic sleeve gastrectomy; MC, high‐protein moderate‐carbohydrate, non‐ketogenic; Med, Mediterranean diet; MWL, medical weight loss; rRNA, ribosomal RNA; RYGB, Roux‐en‐Y‐gastric bypass.

## OBESITY IN SCHIZOPHRENIA

Obesity is particularly prevalent in SZ, with nearly 40%–60% of patients being obese, a rate significantly higher than that in the general population [6, 84, 85]. SZ patients with obesity tend to have poorer cognitive performance and physical condition, as well as shorter life expectancy than their lean counterparts [7]. Thus, obesity is recognized as one of the strongest contributors to adverse psychiatric outcomes in SZ [12]. Previously, our group assessed the cognitive function of patients with SZ using the MATRICS Consensus Cognitive Battery and found that combined metabolic disorders, such as obesity, primarily affect patients' working memory and information processing speed [86], and that these cognitive deficits further have an impact on their social functioning and quality of life [87].

### Factors involved in the pathophysiology of obesity in SZ

Until now, the exact mechanisms underlying the high prevalence of obesity in SZ patients are not fully understood. Among the factors contributing to this high prevalence, the complex interplay between unhealthy lifestyle, adverse effects of antipsychotics, and the inherent pathophysiological mechanisms of SZ are the most cited explanations [84, 88]. SZ patients tend to have unhealthy lifestyles, including alcohol consumption, smoking, poor diet, lack of exercise, and have more difficult social circumstances. In particular, dietary patterns are the key determinant shaping the composition of the gut microbiota. For example, a high‐calorie diet for just 3 days has been reported to result in an increased abundance of *Firmicutes* and decreased abundance of *Bacteriodetes* in human [89]. Likewise, there is additional evidence that the composition of the gut microbiota changes within 24 h of a high‐fat diet [22]. Interestingly, recent studies indicate that the antipsychotic‐induced weight gain may also be attributable to alterations in the gut microbiome [90, 91]. In line with this evidence, preliminary findings in germ‐free mice suggested that olanzapine could induce weight gain and promote a shift towards “obesogenic” microbial profile (i.e., increased *Firmicutes* while decreased *Bacteroides*) only after the colonization of gut microbiota [92]. Similar findings were also observed in antipsychotic‐treated human studies for both children and adults [93, 94]. As risperidone treatment progressed, the gut microbial composition changed dynamically, with a significant positive correlation between the *Firmicutes*/*Bacteroides* ratio and the increase in BMI [94]. Taken together, these data suggest that both dietary patterns and antipsychotic medication may interact with and reshape the microbial profiles, leading to rapid weight gain in patients with SZ. Thus, future research should distinguish those from the innate microbiota of SZ patient, which is crucial for a more thorough understanding of the relationship between gut microbiota and SZ.

Notably, studies also showed that medication‐free SZ patients have impaired fasting glucose, insulin resistance and elevated cortisol levels compared to the healthy population [95, 96]. Moreover, siblings of these patients are at a high risk for MS, which is not dependent on the action of any antipsychotic medication [97]. Similarly, epidemiological research has reported that up to 44.8% of medication‐free SZ patients are obese or overweight, significantly higher than that of healthy controls (36.6%) [98]. This evidence indicates that SZ itself is an independent risk factor for obesity and that it may entail certain pathophysiological processes that have an impact on host metabolism. Conversely, maternal obesity during pregnancy has also been associated with an increased risk of SZ in the offspring [99], further suggesting that the two disorders may share certain pathophysiological mechanisms.

### Gut‐derived inflammation as a potential hub linking SZ and obesity

In recent years, the “gut–brain axis” research has drawn increasing attention, in particular the interaction between gut microbiota and host health. Gut microbial dysbiosis has been observed in both patients with SZ and obesity (Figures 2 and 3), and a perturbed gut–brain axis is closely related to the pathogenesis of the two disorders. As noted in Tables 1 and 2, both obesity and SZ were associated with a decrease in microbial diversity, and both were characterized by a decrease in anti‐inflammatory bacteria with butyrate‐producing capacity (e.g., genera *Faecalibacterium* and *Roseburia*) and an increase in many proinflammatory Gram‐negative bacteria as well as other pathogenic bacteria (e.g., phylum *Proteobacteria* and genus *Anaerococcus*). Since inflammatory processes are involved in the pathophysiology of both obesity and SZ, we hypothesize that gut microbiota‐derived inflammatory signaling may serve as a potential hub linking SZ and obesity, which may be associated with the high prevalence of obesity in SZ.

### Inflammation in obesity

**Figure 4:**
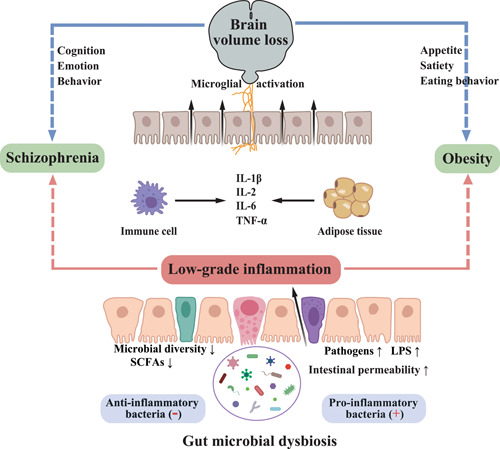
Gut‐derived inflammation as a potential hub linking schizophrenia and obesity. Gut microbial dysbiosis has been observed in both SZ and obesity. It leads to impaired intestinal barrier and thereby increased transport of pathogens and LPS to the circulatory system, resulting in the production of proinflammatory cytokines and elevated inflammation in the circulation. Peripheral cytokine signaling can induce neuroinflammation, leading to structural remodeling of the brain (manifested as brain volume loss), which can lead to changes in cognition, emotion, and behavior, as well as changes in appetite, satiety and eating behavior. IL‐1*β*, interleukin 1*β*; IL‐2, interleukin 2; IL‐6, interleukin 6; LPS, lipopolysaccharide; SCFAs, short‐chain fatty acids; SZ, schizophrenia; TNF‐*α*, tumor necrosis factor *α*.

Obesity is a state of low‐grade inflammation characterized by macrophage infiltration in adipose tissue and release of cytokines, such as tumor necrosis factor *α* (TNF‐*α*) and interleukin 6 (IL‐6) that are produced by macrophages to activate inflammatory signaling and thereby enhance the systemic low‐grade inflammation in obesity [100]. Importantly, this low‐grade inflammation can impair insulin sensitivity causing insulin resistance and hyperinsulinemia, which further exacerbates obesity [101, 102]. In turn, obesity further exacerbates low‐grade inflammation through the secretion of proinflammatory cytokines by adipocytes, thus creating a vicious cycle (Figure 4). Thus, although the causal pathways underpinning the relationship between obesity and inflammation have not been fully identified, obesity and inflammation are certainly causally related to each other.

In clinical study, increased proinflammatory molecules have been consistently observed in obesity. This includes positive correlations between body measurements such as BMI, waist circumference and waist‐to‐hip ratio and C‐reactive protein (CRP) levels [103], and between adipocyte size and levels of TNF‐*α*, IL‐6, and high‐sensitivity CRP [104]. Although most of the studies were based on correlations and did not establish a causal relationship between inflammation and obesity, they did support the likely interplay between obesity and inflammation. In turn, a 10% weight loss over 1 year has also been shown to reduce plasma concentrations of several proinflammatory cytokines in obese women [105]. Moreover, the interaction between inflammation and obesity is also shown by the ability of anti‐inflammatory treatments to improve obesity‐associated insulin resistance. These treatment strategies include IL‐1‐receptor antagonist [106], IL‐1*β*‐neutralizing antibody [107], TNF‐*α* blockade [108], and anti‐inflammatory drug [109]. Altogether, these findings highlight pivotal roles of inflammation in the development of obesity and the opportunities for interventions.

### Inflammation in schizophrenia

Inflammation has long been suggested to play a critical role in the pathogenesis of SZ (Figure 4). Although SZ typically appears in early adulthood, its neuronal disruption is proposed to begin as early as in utero [110]. Indeed, many of the currently proposed early risk factors for SZ are associated with in utero adversities, such as maternal infections and malnutrition during pregnancy, and obstetric complications leading to fetal hypoxia [111, 112, 113]. These hypoxic and infectious factors could stimulate maternal and thereby fetal inflammatory responses, leading to the early “hits” for the pathogenesis of SZ, as the developing brain is highly sensitive to the effects of inflammation. After a series of early hits in utero, SZ may potentially be characterized by a chronic inflammatory state. This evidence includes peripheral and neuroinflammation being increasingly reported in patients with SZ, often already at illness onset. Such studies in SZ have found higher levels of proinflammatory molecules in the cerebrospinal fluid of SZ patients than in controls [114]. Studies of cytokines in the peripheral blood also showed similar results, that is, higher levels of proinflammatory cytokines in both first‐episode SZ and relapsed patients than in healthy controls [115]. In addition, changes in some inflammatory markers may correlate with disease progression. For instance, increased baseline proinflammatory markers, such as IL‐6 and CRP, have been identified as risk factors for poor treatment response at 3‐month follow‐up and worse clinical outcome at 1‐year follow‐up in patients with first‐episode psychosis (FEP) [116, 117].

Notably, inflammation also appears to affect the neurotransmitter system in SZ, which has long been a major focus of research into the neurobiology of SZ. For example, administration of IL‐1*β* at birth affects dopaminergic neurons in adult mice [118], short exposure to IL‐6 reduces the survival of serotonergic neurons in the fetal brain [119], and chronic exposure to interferon‐*α* reduces the release of striatal DA in association with anhedonia‐like behavior [120], a typically negative symptom in chronic SZ. Nevertheless, a critical discussion of these findings should mention that increases in proinflammatory cytokines are not specific to SZ and have been observed in other psychiatric disorders; and that the effects of confounding factors, such as body mass index and medication, should also be considered.

### Gut microbiota and inflammation

The gut microbiota encompasses a diverse community of bacteria that plays an essential role in maintaining host metabolic and immune homeostasis, in part by regulating the permeability of the gut (Figure 4). Certain beneficial bacteria, such as *Faecalibacterium* species, could produce specific enzymes that enable the fermentation of indigestible carbohydrates in dietary fiber into SCFAs, and these SCFAs play a role in maintaining the integrity of the gut wall and inhibiting inflammation [80, 121, 122]. On the other hand, some harmful bacteria, particularly Gram‐negative members, including lipopolysaccharide producers and pathogens, can increase intestinal permeability and facilitate LPS transport to the circulatory system [123]. LPS is an endotoxin in the cell wall of Gram‐negative bacteria, and its binding to Toll‐like receptor‐4 could induce the body's immune inflammatory reaction [124]. Under normal conditions, the gut barrier could minimize the movement of LPS from the bowels into the circulation. Factors such as diet or pathogenic bacteria, may promote a “leaky gut,” where LPS leaves the gut and enters the bloodstream [125, 126]. In response, the body produces proinflammatory cytokines and other mediators that effectively initiate an inflammatory response [127].

This gut microbiota‐derived inflammation has been observed in both obesity and SZ. Recently, a review in *JAMA* pointed out that the depletion of certain anti‐inflammatory butyrate‐producing bacteria and the enrichment of proinflammatory bacteria are shared characteristics of the gut microbiota in patients with psychiatric disorders, including SZ [128]. Similarly, increased level of bacterial LPS has been observed in obesity, and perturbation in the gut microbiota and changes in intestinal permeability have been identified as potential triggers of obesity‐related low‐grade inflammation [102]. Moreover, high levels of peripheral proinflammatory cytokines could further induce neuroinflammation that leads to the neurodegeneration and structural remodeling of the brain, which is consistently observed in both obesity and SZ [129]. Considering the causal effect of inflammation on the development of obesity and SZ, we propose that gut microbiota‐derived inflammation may be a trigger for obesity in SZ, which may also explain the significant risk of SZ among adult offspring born to obese mothers.

### Gut microbiota: A promising therapeutic target for obesity in SZ

**Figure 5:**
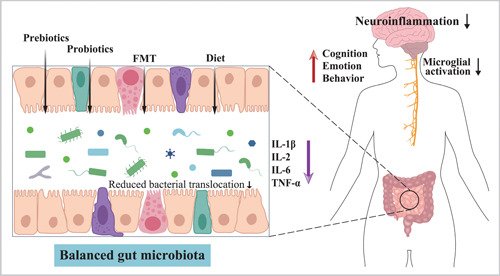
Potential therapeutic options for restoring gut homeostasis and treating obesity in schizophrenia. Administration of probiotics, prebiotics and fecal microbiota transplants leads to a beneficial gut microbiota composition, reduced bacterial translocation and reduced systemic inflammation. The ensuing reduction in proinflammatory cytokines may lead to reduced microglial activation, improved cognition, and reduced appetite and eating behavior. FMT, fecal microbiota transplants; IL‐1*β*, interleukin 1*β*; IL‐2, interleukin 2; IL‐6, interleukin 6; TNF‐*α*, tumor necrosis factor *α*.

Several interventions are potentially able to restore the gut microbial balance to treat obesity in SZ patients (Figure 5). Among them, dietary modification remains the primary intervention. Changes in diet have been reported to explain 57% of the total structural variation in gut microbiota [130], and different dietary components directly shape different gut microbial compositions [131, 132]. For example, fiber is one of the main dietary components that consists of nondigestible carbohydrates. Fermentation of nondigestible carbohydrates by the gut microbiota has been shown to increase the production of SCFAs, which have multiple beneficial effects on host metabolic homeostasis, such as increasing gut microbial diversity and reducing obesity‐associated inflammation [59]. In addition to diet, the use of pre/probiotics is also one of the most widely used strategies to modulate the gut microbiota. In the past 10 years, the beneficial effects of probiotics on weight loss have been extensively demonstrated in individuals with diabetes and obesity [133, 134, 135]. Recently, two randomized clinical trials have also demonstrated for the first time that probiotics plus dietary fiber can effectively reduce the antipsychotic‐induced weight gain and metabolic disturbance in SZ patients [136, 137]. And such favorable effects were associated with an increased abundance of gut microbiota [137]. Similarly, FMT from lean donors has been shown to have beneficial effects on insulin sensitivity in obese individuals [138, 139], accompanied by increased levels of butyrate‐producing bacteria [138]. In addition, Liang et al. also demonstrated that SZ patients with enterotype‐*Prevotella* (characterized by high abundance of *Proteobacteria* and *Firmicutes*) have a higher risk of obesity [140], suggesting that dysbiosis of the gut microbiota may be an essential component in the development of obesity in SZ. Altogether, these findings suggest that the gut microbiota would be a promising therapeutic target for obesity in SZ.

On the other hand, these interventions may also potentially improve the brain volume loss associated with both obesity and SZ (Figure 5). In the general population, weight gain‐related brain volume loss is a major cause of poor cognitive performance in obesity. Similarly, SZ patients with obesity typically experience a worse psychiatric outcome than those with normal weight [12]. On the basis of this evidence, it is tempting to infer that development of obesity may increase the severity of brain volume loss associated with the core symptoms of SZ, leading to a worse disease process. In line with this hypothesis, several studies on SZ patients have shown that obese individuals have more severe brain volume loss in both white matter and gray matter compared with their lean counterparts [10, 11]. More recently, McWhinney et al. [141] replicated the associations between obesity and brain structure in a large multicenter sample from the ENIGMA‐SZ working group and further demonstrated that almost all obesity‐associated brain regions were also associated with SZ. Fortunately, longitudinal studies have confirmed that obesity‐related brain volume loss are reversible through early intervention for weight loss [142, 143]. Thus, these therapeutic approaches targeting the gut microbiota may not only improve obesity and obesity‐associated low‐grade inflammation, but may also ameliorate the brain volume loss associated with clinical, cognitive, and functional outcomes in SZ.

## SUMMARY AND FUTURE DIRECTIONS

In summary, gut microbial dysbiosis has been detected in both SZ and obesity, and perturbations in gut–brain axis, particularly gut‐derived inflammation, are closely related to the pathogenesis of both disorders. This suggests that gut microbiota may be a potential hub linking SZ and obesity, and the microbiota‐derived inflammatory signaling may explain the high prevalence of obesity in SZ. Given that obesity presents a significant challenge in the clinical management of SZ and that gut microbiota is a modifiable component, gut microbiota may represent a novel therapeutic option for treating obesity or improving psychiatric outcomes in SZ patients. Nevertheless, the mechanisms underlying the critical link between obesity and SZ remain to be fully elucidated, and with various psychosocial and other biological factors involved, the role of the gut microbiota requires further exploration and consideration before being adopted as a therapeutic target. Moreover, most current research has concentrated on the role of gut microbiota in maintaining mental health or influencing disease severity, and few studies have been conducted directly on schizophrenic patients with obesity. In addition, there are limitations to the existing studies that require special attention.

In most investigations, the participants' demographic parameters were not sufficiently characterized. Apart from race, age, and gender, additional factors need to be included, such as body mass index, smoking, alcohol consumption, exercise, diet, usage of antibiotics, or other drugs having endocrine or immunomodulatory effects. For instance, eating disorders (e.g., anorexia, bulimia, and binge eating disorder) are particularly prevalent in both SZ and obesity. The eating patterns and food preferences are important determinants of gut microbiota. However, it is unclear how eating disorders affect the specific gut microbiota in these patients. Additionally, existing evidence have shown that antipsychotic medications have a major impact on the gut microbiota and thereby dramatically raise the risk of obesity [144]. Thus, future studies would benefit from this consideration. Similarly, the clinical features of the disease are not well evaluated, including the age of onset, duration of illness, classification and severity of symptoms, risk of suicide, and the presence of other chronic conditions. For example, most previous studies have not further explored potential differences in gut microbial diversity and composition between individuals with predominantly positive symptoms in SZ and those with predominantly negative symptoms. Given that those patients with the two symptom clusters present with distinctly diverse clinical presentations, it is necessary to clarify the inherent connection between these clinical features and specific microbial taxa. Moreover, most previous studies had a cross‐sectional design with small sample sizes, and there was considerable heterogeneity in fecal sample collection, storage, and analysis methods across studies, which may affect the outcomes as well [145]. To assure sample and data quality, a systematic protocol for handling stool samples should be developed.

The interaction between gut microbiota and host health is dynamic and variable. To enhance comparison between studies and facilitate meta‐analyses, it is crucial to accurately identify and profile personal and disease‐related characteristics linked with gut microbiota. More large‐scale and multiomics studies combining host genomics, microbiomics, metabolomics, and brain connectomics are also required to fully understand the complex operating mechanisms of the MGB axis. Although studies on the gut microbiota in SZ have recently accumulated, research on gut microbiota in SZ patients with obesity and its relationship with brain imaging features is still in its infancy. Future research should therefore focus more on changes in the gut microbiota before and after the development of obesity in SZ patients and assess how the dynamics of these microbiota–host interactions and the resulting alterations in gut–brain communication, such as gut‐derived metabolites and inflammatory markers, further come to influence brain structure and function. In addition, clinical translation of these findings is needed to consider interventions targeting gut microbiota as potential therapies to improve the clinical outcomes of SZ patients. This will lay a solid theoretical platform for revealing the pathophysiology of obesity in SZ patients and pave the way for the creation of fresh methods for the prevention and treatment of obesity in SZ patients.

## AUTHOR CONTRIBUTIONS

Xiaoli Wu and Liwei Xie designed this frame of the manuscript. Hui Wu drafted the manuscript. Hui Wu, Yaxi Liu, Jie Wang, Shengyun Chen, and Liwei Xie edited the manuscript.

## CONFLICT OF INTEREST STATEMENT

The authors declare no conflict of interest.

## Data Availability

Supplementary materials (figures, tables, scripts, graphical abstract, slides, videos, Chinese translated version, and update materials) may be found in the online DOI or iMeta Science http://www.imeta.science/.
